# Preliminary identification of psychometric cut-off points for the SQoL-CHD: a cross-sectional study based on LPA and ROC analyses in a Chinese clinical sample

**DOI:** 10.3389/fpubh.2026.1855052

**Published:** 2026-05-29

**Authors:** Weihong Yang, Fengpei Zhang, Yachai Li, Ruijuan Fan

**Affiliations:** 1 Nursing Department, The First Affiliated Hospital of Xinxiang Medical University, Xinxiang, China; 2 Department of Cardiovascular Ward, The First Affiliated Hospital of Xinxiang Medical University, Xinxiang, China; 3 Department of Oncology Ward, The First Affiliated Hospital of Xinxiang Medical University, Xinxiang, China

**Keywords:** coronary heart disease patient, cut-off point, LPA, mental health, ROC, sexual quality of life

## Abstract

**Objective:**

To determine psychometric cut-off points for the male and female versions of the Sexual Quality of Life Questionnaire for Coronary Heart Disease (SQoL-CHD), and to validate their effectiveness in distinguishing patients with low vs. high sexual quality of life, as well as their association with psychological distress.

**Methods:**

A cross-sectional study using convenience sampling was conducted among 1,368 patients with coronary heart disease from a large general hospital in Henan Province, China between March and December 2024. The sexual quality of life questionnaire for patients with coronary heart disease (male version and female version), the Generalized Anxiety Scale (GAD-7) and the Patient Health Questionnaire-9 (PHQ-9) were used for measurement. LPA was used to identify latent classes of sexual quality of life, and then ROC analysis was used to determine the optimal cut-off point, and its validity was verified by anxiety and depression levels.

**Results:**

LPA results show, the sexual quality of life for both male and female patients was classified into three latent classes, demonstrating high classification accuracy (Entropy > 0.96). ROC analysis determined that the optimal cut-off point for the female version of the questionnaire was 58 (sensitivity 96.8%, specificity 96.6%, AUC = 0.997), and that for the male version was 68 (sensitivity 98.5%, specificity 99.0%, AUC = 0.999). The cut-off point validation results showed that the patients in the low sexual quality of life group had significantly higher scores in sexual physiology, sexual psychology, sexual cognition dimensions, as well as anxiety and depression levels than those in the high sexual quality of life group, and male patients had a higher risk of anxiety and depression.

**Conclusion:**

The SQoL-CHD questionnaire cut-off points demonstrate strong psychometric properties and are effective for identifying CHD patients at risk of low sexual quality of life and higher levels of anxiety and depression symptoms, supporting their use in targeted clinical screening and intervention.

## Introduction

Coronary Heart Disease (CHD) is one of the leading causes of death and disability worldwide, with its chronic progression and acute attacks having a significant impact on patients' quality of life (1). As the incidence of CHD increases year by year and shows a trend of becoming younger (2), The Sexual Quality of Life (SQoL), a psychological dimension reflecting an individual's sense of intimacy and emotional connection, has increasingly become a focus in chronic disease management (3, 4). The quality of sexual life is not only an important component of patients' quality of life but also a key psychological indicator for measuring individual subjective well-being, self-efficacy, and adaptation to chronic illness (5, 6). Studies have shown that coronary heart disease can affect patients' sexual life, leading to a decrease in sexual satisfaction (7), this phenomenon involves multiple factors, including physiological, psychological, and socio-cultural factors (8). From a physiological perspective, patients with coronary heart disease are troubled by symptoms such as decreased heart function, fatigue, and angina. Certain therapeutic drugs, such as beta-blockers, statins, antihypertensive drugs, and thiazide diuretics, can cause sexual dysfunction or sexual desire, thus affecting the patient's sexual quality of life (9–11). Although drug therapy can exert certain positive effects by improving cardiovascular conditions or directly treating sexual dysfunction (such as with phosphodiesterase-5 inhibitors), the use of such drugs in patients with cardiovascular disease requires careful assessment of cardiovascular risks and related contraindications. Therefore, they still have certain limitations in clinical practice. On a psychological level, symptoms of anxiety and depression, as well as concerns about sexual activity potentially triggering cardiac events, may negatively affect patients' sexual quality of life (11–13). At the sociocultural level, factors such as marital relationships and attitudes toward sex also have a significant impact on the sexual quality of life (14).

Although there are studies on sexual quality of life in patients with coronary heart disease, there are still some limitations. Existing research has mostly focused on the discussion of sexual dysfunction (15, 16), While ignoring the independent value of sexual quality of life as a psycho-social experience. In fact, the sexual quality of life and sexual function are two related but different concepts: sexual function mainly focuses on physiological aspects such as libido, sexual arousal, erectile function, sexual intercourse and orgasm (17), while the sexual quality of life emphasizes the patient's subjective feelings, emotional experience and overall satisfaction with sexual life. Decreased sexual quality of life in patients with CHD may be accompanied by sexual dysfunction (18, 19), but is not limited to this. Some patients with normal sexual function may still experience low sexual satisfaction due to psychological factors, relationship or social pressure (11–13). Existing research has mostly focused on the physiological mechanisms and treatments of sexual dysfunction (20), ignoring the subjective experience of sexual quality, which is more important for mental health and overall quality of life. To make up for this deficiency, our research group developed and validated the Sexual Life Quality Questionnaire for Patients with CHD (21), which measures the quality of sexual life from three dimensions: sexual physiology, sexual psychology, and sexual cognition. However, this tool has not yet clearly defined a quantitative threshold between “high sexual life quality” and “low sexual life quality,” limiting its application value in psychological screening and clinical intervention.

Determining cut-off points is of great significance in epidemiological research and clinical interventions (22). Reasonable cut-off points can divide the population into different risk levels, help medical staff identify high-risk patients, and then develop personalized intervention plans. Usually, clinical outcomes are regarded as the gold standard for evaluating scale performance and selecting cut-off points for assessment tools. However, in the absence of clinical outcome data, statistical methods such as Latent Profile Analysis (LPA) and Receiver Operating Characteristic (ROC) curves have been widely used in research to construct data-driven psychometric thresholds (22, 23). LPA can classify samples by identifying the latent classes of the data, while ROC evaluates the ability of the cut-off point to predict the actual state (22). The combination of both not only enhances the statistical validity of threshold setting but also strengthens its psychological interpretability.

Clarifying the cut-off point of the sexual quality of life questionnaire for patients with coronary heart disease has important theoretical value and practical significance. On the one hand, as a comprehensive indicator reflecting the patient's overall health status and psychosocial adaptability, the decline in sexual quality may reveal weak links in disease management or unrecognized psychological needs of patients. Reasonable setting of the cut-off point will help medical staff and mental health practitioners to identify high-risk groups with low quality of sexual life at an early stage and implement individualized intervention measures. On the other hand, the establishment of the cut-off point provides an operational basis for the promotion and application of the sexual quality of life assessment tool for patients with coronary heart disease in the clinical and mental health fields, and improves its interpretability and practical application value. In addition, traditional culture and the sensitivity of sexual topics make many patients reluctant to actively discuss sexual life issues, and medical staff do not often take the initiative to communicate about them (24). In this context, the setting of cut-off points provides an intuitive reference standard for patients and medical staff, helps reduce the difficulty of communication, and promotes the timely identification and resolution of problems. Therefore, this study will determine the cut-off point for the male and female versions of the sexual quality of life questionnaire for patients with CHD based on LPA and ROC analysis methods, and combine variables such as mental health (anxiety, depression) to comprehensively explore the factors affecting the quality of sexual life and validate the appropriateness of the identified cut-off points. This research provides clear criteria for the screening and intervention of sexual quality of life among patients with CHD, thereby offering scientific evidence for clinical practice and promoting the widespread application of assessment tools.

## Methods

### Study design and participants

This study was a cross-sectional study and relevant data were collected through a questionnaire survey. A convenient sampling method was used to select patients with CHD in the cardiovascular ward and cardiovascular outpatient clinic of a hospital in Henan Province, China from March to December 2024. Inclusion criteria: Aged ≥18 years and with a stable sexual partner; Had normal sexual life prior to illness; Patients diagnosed with CHD and with stable condition, with functional status required to be consistent with New York Heart Association (NYHA) class I-II; Able to understand and complete the questionnaire; Informed consent and voluntary participation. Exclusion criteria: CHD combined with chronic heart failure; Mental or psychological illness; Other serious acute or chronic diseases. To ensure the statistical power of the study and the reliability of the results, this study estimated the sample size based on the sample size calculation formula of ROC analysis (25). ROC analysis was used to determine the optimal cut-off point of the Sexual Quality of Life Questionnaire in patients with CHD, and the sample size was calculated based on the expected the area under the curve (AUC) and the allowable margin of error. The formula used is: *n* = ${\left(Z_{\alpha /2}+Z_{\beta }\right)}^{2}\cdot p\left(1--p\right)/{\left(\delta \right)}^{2}$. Among them: *Z*_α/2_ is 1.96, corresponding to α = 0.05, *Z*_β_ is 0.84, corresponding to 80% power; *p* is assumed to be 0.75; δ is set to 0.05. The sample size for this study was 588 cases. Taking into account the 5% loss to follow-up, the sample size required for this study was at least 617 cases. This sample size can not only ensure that the AUC estimation of ROC analysis has sufficient accuracy, but also meet the requirements of LPA on the sample size, to ensure the stability of the model and the reliability of classification (26, 27). This study was reported using the STROBE Report List to ensure research transparency and report quality.

## Measures

### Demographic characteristics of participants

It includes the basic information of the patient, including age, education level, occupation, income level, etc., and disease-related information, such as the course of coronary heart disease, treatment methods, etc.

### Sexual quality of life questionnaire for patients with coronary heart disease (SQoL-CHD)

This scale was developed by our research team and includes three dimensions: sexual physiology, sexual psychology, and sexual cognition. It is divided into two versions: male versions and female versions (21). The male version consisted of 20 items and its Cronbach's α coefficient was 0.942, the Cronbach's α coefficients of each dimension ranged from 0.733 to 0.925. The female version consisted of 17 items, and its Cronbach's α coefficient was 0.952, and Cronbach's α coefficient of each dimension was 0.710 ~ 0.954. The questionnaire was scored at Likert 5 level, according to “strongly disagree, disagree, general, agree, strongly agree” 5 ranking. The higher the questionnaire score, the poor quality of sexual life.

### Generalized anxiety disorder scale-7 (GAD-7)

A concise self-assessment scale for anxiety symptoms, developed by Spitzer et al. based on the diagnostic criteria of the and Statistical Manual of Mental Disorders, 4th Edition (DSM-IV), is used to evaluate the frequency of anxiety symptoms over the past two weeks (28). The scale consists of 7 items and uses a 4-point Likert scoring method: “Not at all” = 0 points, “Several days” = 1 point, “More than half the days” = 2 points, and “Nearly every day” = 3 points. The score ranges from 0 to 21, with 0–4 indicating no anxiety and ≥5 indicating the presence of anxiety. The correlation coefficient between each item and the total score of the Chinese version GAD-7 was 0734–0.820, the correlation coefficient between each item was 0.483–0.712, and the Cronbach 'a coefficient was 0.90.

### Patient health questionnaire-9 (PHQ-9)

This scale is a commonly used screening scale in clinical depression diagnosis and general population survey research (29). The PHQ-9 has 9 items, which are designed to understand how much time you have been bothered by 9 problems in the past 2 weeks, including lack of interest and low mood. The score range of this scale is 0 to 27, with 0 to 4 points indicating no depression, 5 to 9 points indicating mild depression, and ≥10 points indicating moderate to severe depression. The internal consistency coefficient of PHQ-9 was 0.857, the correlation coefficient between items was 0.236–0.718, and the correlation coefficient between items and the total score of the scale was 0.588–0.784.

### Statistical analysis

Statistical analysis was performed using R 4.4.3, and the significance level was set at 0.05.

**Descriptive analysis:** In the descriptive analysis of demographic characteristics and scores of the main measurement variables, continuous variables were expressed as mean ± standard deviation, and categorical variables were expressed as frequency and percentage.

**LPA:** To identify the latent classes of sexual quality of life among patients with CHD, LPA was conducted based on the scores of the male and female versions of SQoL-CHD, with model fitting performed using R 4.4.3. The model fit was evaluated by gradually adding latent classes (*k* = 1, 2, 3, …) and based on the Akaike Information Criterion (AIC), the Bayesian Information Criterion (BIC), the sample-size adjusted BIC (ssaBIC), and Entropy (30)^.^ Lower AIC and BIC values indicate better model fit (31, 32); an Entropy value closer to 1 indicates clearer classification (33). The Lo-Mendell-Rubin test (LMRT) and the Bootstrap Likelihood Ratio Test (BLRT) are used at the same time. If *p* < 0.05, it means that the model containing k classes is better than the model containing *k*-1 classes (26, 27). In addition, considering the clinical significance and the sample size of each profile, the sample size is required to be >5% (22, 34, 35). Cohen's *d* was calculated as a measure of effect size to verify the accuracy of the classification. Cohen's *d* values of 0.2, 0.5, and 0.8 represent small, medium, and large effects, respectively (36).

**ROC analysis:** On the basis of the classification, ROC analysis was used to determine the optimal cut-off point for sexual quality of life. The classification performance of the scores from different versions of SQoL-CHD was calculated using the pROC package in R 4.4.3. Indicators such as Sensitivity, Specificity, Positive Predictive Value (PPV) and Negative Predictive Value (NPV) are used to evaluate the classification performance of the cut-off point. The classification accuracy was evaluated using the AUC, with an AUC value closer to 1 indicating higher classification performance (37). Youden Index was finally selected to determine the best cut-off point for the two versions of SQoL-CHD.

**The validity analysis of the optimal cut-off point:** Independent Samples *t*-test, analysis of variance and odds ratios (ORs) were used to compare the differences in anxiety and depression levels between the high and low sexual quality of life groups based on the optimal cut-off point, and to verify the clinical significance of the questionnaire cut-off point.

### Ethics

Before the study began, the purpose, methods, and significance of the study were explained in detail to all participants. Inform participants that they have the right to withdraw from the study at any time without facing any adverse consequences. All research data are collected and stored anonymously to ensure that the personal privacy of participants is not disclosed. After fully understanding the content of the study, the participants voluntarily signed the informed consent. This study was approved by the Ethics Committee of the First Affiliated Hospital of Xinxiang Medical University (No: EC-022-005) and was conducted in accordance with the principles outlined in the Declaration of Helsinki.

## Result

### Demographic characteristics

A total of 1,368 patients were included in this study, including 683 male patients, accounting for 49.9% of the total number, and 685 female patients, accounting for 50.1% of the total number. The average age of males was 54.93 ± 9.6 years, and the average age of females was 56.83 ± 7.54 years. More than half of the male and female patients had only junior high school education, accounting for 59.2% and 59.3% respectively. 43.3% of males and 44.1% of females had a disease duration of >6 months (Table 1).

**Table 1:** Sociodemographic characteristics of participants.

Characteristics	Items	Male (*n* = 683)	Female (*n* =685)
		Percentage (%)	Percentage (%)
Age	≤ 30	0.6	0
	31–40	7.8	0
	41–50	22.0	20.1
	51–60	42.2	54.2
	61–70	24.2	21.2
	≥71	3.4	4.5
Education level	Junior high school or below	59.2	59.3
	senior high school	24.3	19.4
	Junior college	9.5	10.1
	Undergraduate college	7.0	11.2
Income	<3,000	44.1	54.2
	3,000–5,000	42.3	35.6
	5,000–10,000	11.6	8.6
	>10,000	2.0	1.6
Course of disease(years)	<0.5	43.3	44.1
	0.5–1	18.7	22.2
	1–2	13.9	14.0
	2–3	10.7	7.4
	>3	13.3	12.3
PCI	No	60.5	73.4
	Yes	39.5	26.6

### LPA results

**Figure 1:**
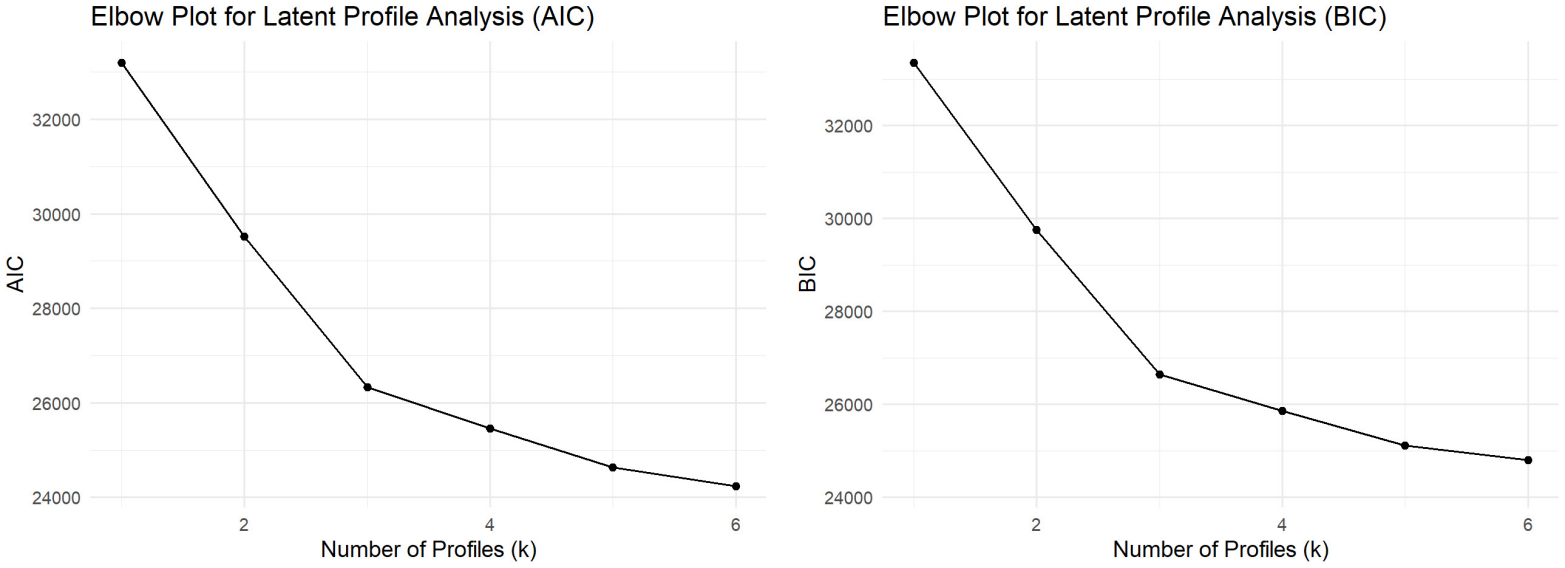
Screen plot of AIC and BIC (female version). AIC, Akaike Information Criterion, BIC, Bayesian information criterion.

**Figure 2:**
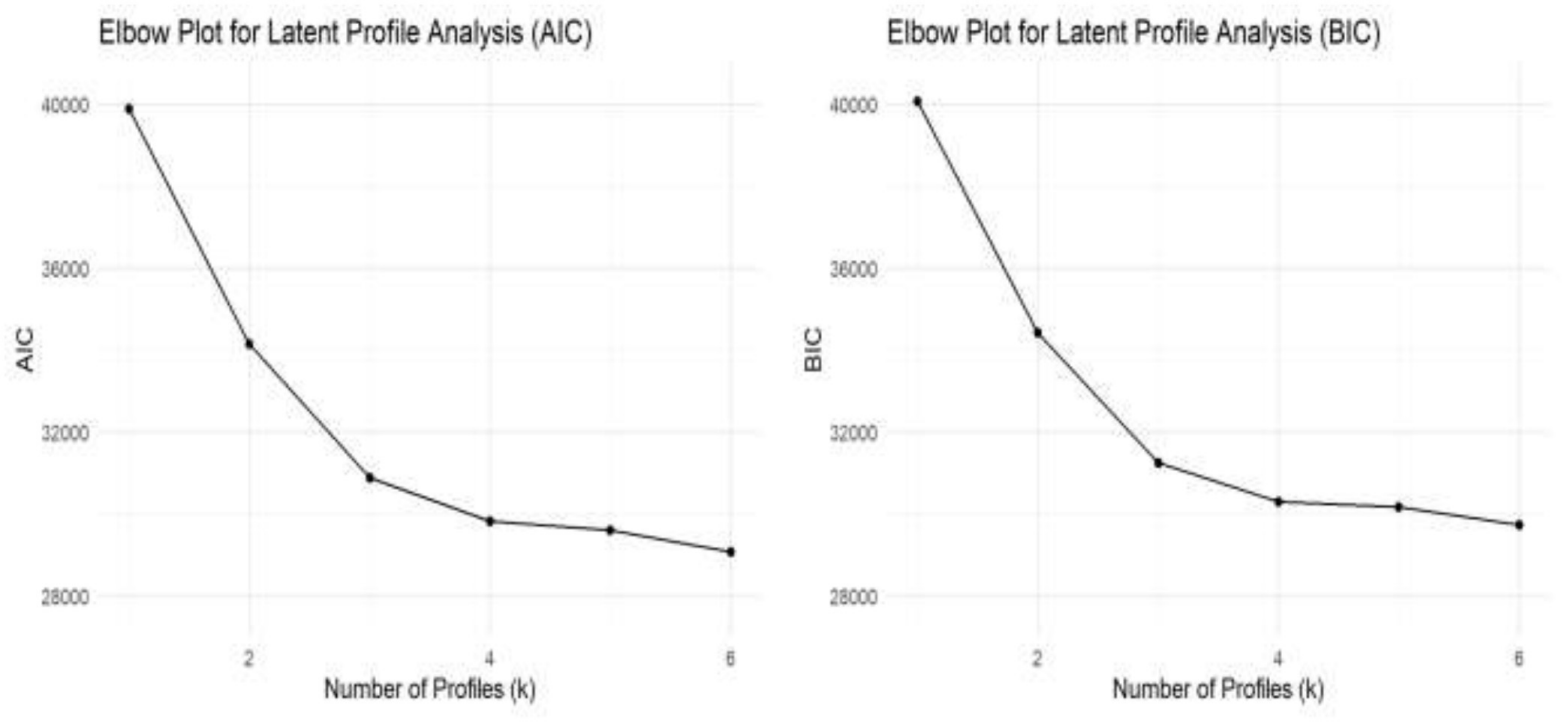
Scree plot of AIC and BIC (male version). AIC, akaike information criterion, BIC, Bayesian information criterion.

Tables 2, 3 show the latent profile analysis (LPA) model fitting indicators of 1 to 6 profiles of the female and male versions of the Sexual Quality of Life Questionnaire for Patients with CHD, respectively. The optimal number of latent profiles for both was determined by combining the scree plot analysis. As the number of profiles increased from 1 to 6, the LogLik, AIC, BIC, G^2^/LL, and ssaBIC values of the female and male versions gradually decreased, indicating a continuous improvement in model fit. The entropy value of the female version reached 0.962 at 3 profiles, and the male version reached the highest (0.969) at 3 profiles. The pLMR and pBLRT of the two versions were significant at 3 profiles (*p* < 0.05), indicating that the 3-profile model had good discrimination and significance in both versions of the questionnaire. In addition, the AIC and BIC scree plots for the female version (Figure 1) and the male version (Figure 2) both show a significant decline as the number of profiles increases from 1 to 3, with a smaller decline from 3 to 6. Notably, a clear “elbow point” is observed at 3 profiles in both versions, further supporting the selection of the 3-profile model as the optimal choice. Although the Entropy value in the female version of the model fit index was slightly lower in the 3-profile model than in the model with a higher number of profiles, the 3-profile model were finally selected after comprehensive consideration of other fit indices. Based on the comprehensive analysis of the above model fit indices and scree plots, the optimal number of latent profiles for both the female and male versions of SQoL-CHD was determined to be 3.

**Table 2:** Fit indices of latent profile analysis (female version).

Classes	LogLik	AIC	BIC	G^2^/LL	ssaBIC	Entropy	pLMR	pBLRT	Profile_probability
1	−16,564.70561	33,197.41121	33,351.41146	33,129.41121	33,351.41146	-	-	-	-
2	−14,708.08251	29,520.16501	29,755.69479	29,416.16501	29,752.43008	0.941	<0.05	<0.05	30.8/69.2
3	−13,093.15806	26,326.31612	26,643.37544	26,186.31612	26,636.84602	0.962	<0.05	<0.05	57.66/19.56/22.77
4	−12,638.35519	25,452.71037	25,851.29923	25,276.71037	25,841.5051	0.967	<0.05	<0.05	18.54/53.58/6.42/21.46
5	−12,208.81127	24,629.62253	25,109.74093	24,417.62253	25,096.68209	0.969	<0.05	<0.05	49.2/18.25/6.42/3.8/22.34
6	−11,994.98	24,237.95999	24,799.60793	23,989.95999	24,783.28438	0.976	<0.05	<0.05	5.26/47.74/18.1/6.42/3.65/18.83

**Table 3:** Fit indices of latent profile analysis (male version).

Classes	LogLik	AIC	BIC	G^2^/LL	aBIC	Entropy	pLMR	pBLRT	Profile_Probability
1	−19,903.69105	39,887.3821	40,068.44189	39,807.3821	40,068.44189	-	-	-	-
2	−17,016.58053	34,155.16106	34,431.27725	34,033.16106	34,428.014	0.972	<0.05	<0.05	75.26/24.74
3	−15,360.22816	30,884.45632	31,255.62889	30,720.45632	31,249.1024	0.969	<0.05	<0.05	29.72/16.25/54.03
4	−14,810.38222	29,826.76445	30,292.99342	29,620.76445	30,283.20368	0.965	<0.05	<0.05	48.02/8.49/16.4/27.09
5	−14,678.38628	29,604.77257	30,166.05793	29,356.77257	30,153.00494	0.955	<0.05	<0.05	44.8/8.05/8.05/26.65/12.45
6	−14,389.09749	29,068.19497	29,724.53673	28,778.19497	29,708.22049	0.943	<0.05	<0.05	8.05/18.3/7.91/38.51/15.81/11.42

**Figure 3:**
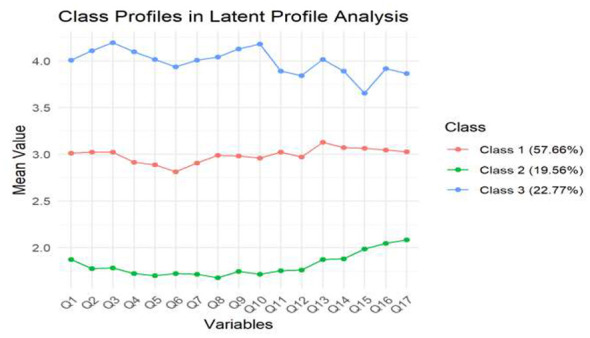
The category characteristics of the three profiles (female version).

**Figure 4:**
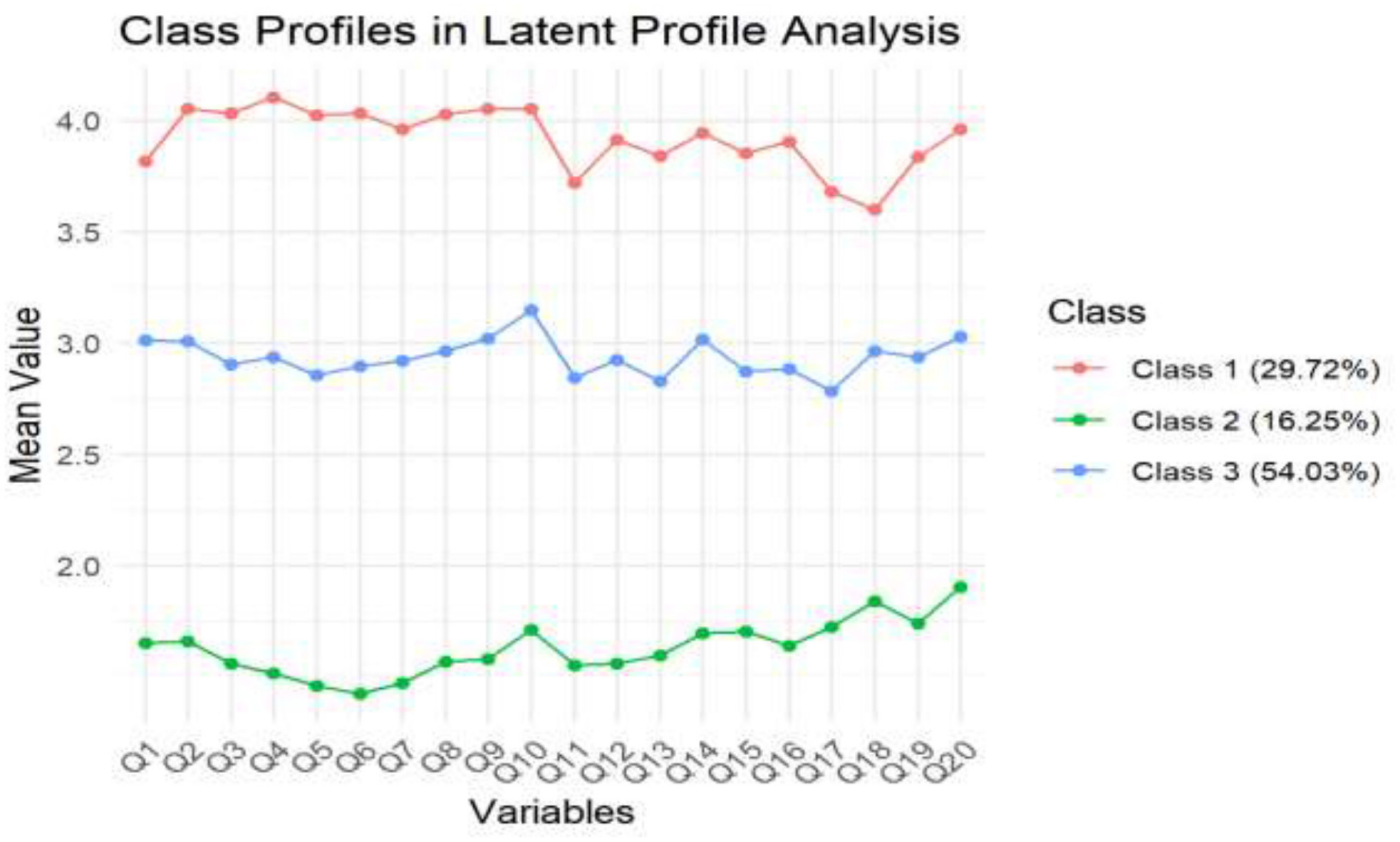
The category characteristics of the three profiles (male version).

According to the 3-class model of latent profile analysis (Figures 3, 4), female participants were divided into low, medium, and high groups, accounting for 19.56%, 57.66%, and 22.77% of female participants, respectively (Table 2). Male participants were also divided into low, medium and high groups, accounting for 16.25%, 54.03% and 29.72% of male participants respectively (Table 3). Table 4 presents the means, standard deviations, and Cohen's *d* values for pairwise comparisons of the three sections of the female and male versions of SQoL-CHD. The results showed that the pairwise comparisons between the three profiles of male and female participants showed large effect sizes (the absolute values of Cohen's *d* were all >0.8). Specifically, among male participants, profile 1 and profile 2 (*d*_1 − 2_ = 3.00), profile 2 and profile 3 (*d*_2 − 3_ = −1.88), and profile 1 and profile 3 (*d*_1 − 3_ = 1.36); among female participants, profile 1 and profile 2 (*d*_1 − 2_ = 1.68), profile 2 and profile 3 (*d*_2 − 3_ = −2.90), and profile 1 and profile 3 (*d*_1 − 3_ = −1.40). These large effect sizes suggest that the characteristics of sexual quality of life differ significantly across profiles, reflecting the practical significance and discriminatory power of the latent profile classification. In the subsequent ROC analysis, participants in the high-quality group were defined as the “low sexual quality of life group”, and the remaining participants (the medium and low groups) were defined as the “high sexual quality of life group”.

**Table 4:** The mean, standard deviation, and Cohen's *d* value of each profile.

Profile	Mean±SD		Class pair	Cohen's *d*	
	Male	Female		Male	Female
Profile 1	3.92 ± 0.78	2.99 ± 0.69	*d*_1 − 2_	3.00	1.68
Profile 2	1.63 ± 0.73	1.81 ± 0.73	*d*_2 − 3_	−1.88	−2.90
Profile 3	2.94 ± 0.69	3.99 ± 0.77	*d*_1 − 3_	1.36	−1.40

Profile 1 is the high group, Profile 2 is the low group, and Profile 3 is the medium group (Male version). Profile 1 is the medium group, Profile 2 is the low group, and Profile 3 is the high group (Female version).

### ROC analysis results

**Figure 5:**
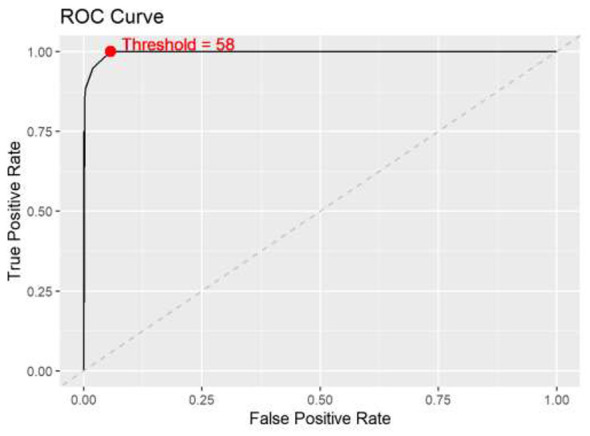
The ROC curve for distinguishing between high and low quality of life in coronary heart disease (female version).

**Figure 6:**
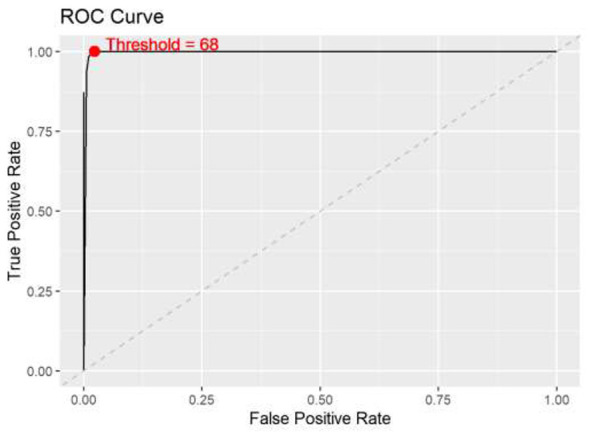
The ROC curve for distinguishing between high and low quality of life in coronary heart disease (male version).

In the ROC analysis, the high group (Profile 3) of the female version of SQoL-CHD was defined as the “low sexual quality of life group,” while the medium group (Profile 1) and low group (Profile 2) were defined as the “high sexual quality of life group.” The high group (profile 1) of the male version of the questionnaire was defined as the “low sexual quality of life group”, and the medium group (profile 3) and the low group (profile 2) were defined as the “high sexual quality of life group”. Based on ROC analysis, the true positive, true negative, false positive, false negative, sensitivity, specificity, positive predictive value, negative predictive value, accuracy and Youden index were determined for different cut-off points. Table 5 shows that when the cut-off point of the female version of the questionnaire was 58, the sensitivity was 96.8%, the specificity was 96.6%, the PPV was 83.9%, the NPV was 100%, the accuracy was 95.6%, the Youden index was 0.943, and AUC was 0.997 (95% CI: 0.995 ~ 0.999; P <0.001) (Figure 5), which shows that this cut-off point achieved a good balance between sensitivity and specificity and had good clinical value and accuracy in screening patients with low sexual quality of life. In contrast, when the cut-off point was 57, the sensitivity was 100%, the specificity was 94.3%, the PPV was 81.7%, the NPV was 100%, the accuracy was 94.9%, and the Youden index was 0.934. Although the sensitivity was higher, the specificity and Youden index were slightly lower. As the cut-off point increased from 53 to 62, the sensitivity gradually decreased (from 100% to 75.0%), although the specificity gradually increased (from 84.3% to 100%), but the sensitivity was low. Therefore, based on comprehensive indicators such as sensitivity, specificity and Youden index, the optimal cut-off point for the female version of SQoL-CHD was ultimately determined to be 58. Table 6 shows that when the cut-off point of the male version of the questionnaire was 68, the sensitivity was 98.5%, the specificity was 99.0%, the PPV was 94.9%, the NPV was 100%, the accuracy was 98.4%, the Youden index was 0.977, and AUC was 0.999 (95% CI: 0.998 ~ 0.999; P <0.001) (Figure 6), indicating that this cut-off point achieved a good balance between sensitivity and specificity. In contrast, when the cut-off point was 67, the sensitivity was 100%, the specificity was 97.7%, the PPV was 90.2%, the NPV was 100%, the accuracy was 96.8%, and the Youden index was 0.954. Although the sensitivity is higher, the specificity, accuracy and Youden index are slightly lower. As the cut-off point increased from 61 to 72, sensitivity gradually decreased (from 100% to 76.8%), while specificity gradually increased (from 81.5% to 100%), and the Youden index initially increased from 0.756 to a peak of 0.977 at a cut-off point of 68, and then gradually declined to 0.803. Therefore, based on comprehensive indicators such as sensitivity, specificity and Youden index, the optimal cut-off point for the male version of SQoL-CHD was ultimately determined to be 68.

**Table 5:** Cut-off point of the sexual life quality questionnaire for patients with coronary heart disease (female version).

Cut-off point	True positive	False negative	True negative	False positive	Sensitivity (%)	Specificity (%)	PPV (%)	NPV (%)	Accuracy (%)	Youden index
53	156	0	420	109	100	84.3	58.9	100	84.1	0.794
54	156	0	446	83	100	87.1	65.3	100	87.9	0.843
55	156	0	461	68	100	89.8	69.6	100	90.1	0.871
56	156	0	475	54	100	93.4	74.3	100	92.1	0.898
57	156	0	494	35	100	94.3	81.7	100	94.9	0.934
58	156	0	499	30	96.8	96.6	83.9	100	95.6	0.943
59	151	5	511	18	94.9	98.1	89.3	99.0	96.6	0.934
60	148	8	519	10	88.5	99.6	93.7	98.5	97.4	0.930
61	138	18	527	2	85.3	99.8	98.6	96.7	97.1	0.881
62	133	23	528	1	75.0	100	99.3	95.8	96.5	0.851

**Table 6:** Cut-off point of the sexual life quality questionnaire for patients with coronary heart disease (male version).

Cut-off point	True positive	False negative	True negative	False positive	Sensitivity (%)	Specificity (%)	PPV (%)	NPV (%)	Accuracy (%)	Youden index
61	203	0	363	117	100	81.5	63.4	100	82.9	0.756
62	203	0	391	89	100	84.0	69.5	100	87.0	0.815
63	203	0	403	77	100	88.3	72.5	100	88.7	0.840
64	203	0	424	56	100	90.4	78.4	100	91.8	0.883
65	203	0	434	46	100	92.3	81.5	100	93.3	0.904
66	203	0	443	37	100	95.4	84.6	100	94.6	0.923
67	203	0	458	22	100	97.7	90.2	100	96.8	0.954
68	203	0	469	11	98.5	99.0	94.9	100	98.4	0.977
69	200	3	475	5	93.6	99.4	97.6	99.4	98.8	0.975
70	190	13	477	3	87.2	100	98.4	97.3	97.7	0.930
71	177	26	480	0	80.3	100	100	94.9	96.2	0.872
72	163	40	480	0	76.8	100	100	92.3	94.1	0.803

### The validity analysis of the optimal cut-off point

First, to verify the effectiveness of the optimal cut-off points determined for the female and male versions of the SQoL-CHD (58 and 68 points, respectively) in distinguishing between individuals with high and low sexual quality of life, we compared the scores of different groups across each dimension of the questionnaire. Table 7 shows that the low sexual quality of life group (female scores ≥ 58, male scores ≥ 68) had significantly higher scores in the dimensions of sexual physiology, sexual psychology, and sexual cognition compared to the high sexual quality of life group (female scores <58, male scores <68). The scores of each dimension in the female group ranged from 26.02 to 39.52, and those in the male group ranged from 26.24 to 39.83, and the differences were statistically significant (*p* < 0.05). The corresponding Cohen's d values showed a large effect size, ranging from 1.53 to 2.23 for females and 1.64 to 2.13 for males, indicating that the cut-off points have strong discriminative power.

**Table 7:** Comparison of dimensional scores between the low sexual quality of life group and the high sexual quality of life group.

Dimensions	Male version (*n* = 683) (Mean ± SD)				Female version(*n* =685) (Mean ± SD)			
	Low sexual quality of life group(≥68)	High sexual quality of life group(<68)	*t*	Cohen's *d*	Low sexual quality of life group (≥58)	High sexual quality of life group(<58)	*t*	Cohen's *d*
Sexual physiology	39.83 ± 4.40	26.24 ± 7.08	30.57^**^	2.13	39.52 ± 4.98	26.02 ± 6.39	29.11^**^	2.23
Sexual psychology	23.08 ± 3.26	15.47 ± 4.21	25.72^**^	1.93	15.51 ± 2.86	10.71 ± 2.84	19.59^**^	1.69
Sexual cognition	15.02 ± 2.20	10.59 ± 2.89	22.01^**^	1.64	11.33 ± 1.90	8.22 ± 2.08	18.56^**^	1.53

Sexual physiology, sexual psychology and sexual cognition are the three dimensions of the questionnaire respectively.

^**^p < 0.05.

To further verify the external validity of the LPA and ROC analysis results, the anxiety and depression levels of people with different levels of sexual quality of life were compared. Table 8 shows that among the three latent profiles identified by LPA, as the sexual quality of life scores of patients with coronary heart disease increased (i.e., the sexual quality of life decreased), the GAD-7 and PHQ-9 scores showed a gradual upward trend, and the differences were significant (*p* < 0.05). Specifically, among females, the mean GAD-7 score increased from 12.04 to 13.11, and the PHQ-9 score increased from 14.44 to 15.61. Among males, the GAD-7 score rose from 9.65 to 13.42, and the PHQ-9 score increased from 11.73 to 16.31. These differences were statistically significant (*p* < 0.05). In addition, Table 9 shows that the average GAD-7 and PHQ-9 scores of the female low sexual quality of life group were 13.80 and 16.62, respectively, which were significantly higher than the 12.79 and 15.22 of the high sexual quality of life group (*p* < 0.05). The corresponding OR values were 1.05 (95% CI: 1.01–1.09) and 1.05 (95% CI: 1.02–1.08), respectively, indicating that the lower the quality of sexual life, the higher the risk of anxiety and depression. The male group also showed a similar trend. The average GAD-7 and PHQ-9 scores of the low sexual quality of life group were 13.44 and 16.24, respectively, which were higher than the 11.67 and 14.11 of the high sexual quality of life group. The difference was statistically significant (*p* < 0.05). The OR values were 1.09 (95% CI: 1.05–1.13) and 1.06 (95% CI: 1.04–1.09), respectively, further supporting that the set cut-off point has good discriminative ability.

**Table 8:** Comparison of external factors among the three latent profiles.

Variables	Male version				Female version			
	Profile (Mean ± SD)			*F*	Profile(Mean±SD)			*F*
	Low score group	Medium score group	High score group		Low score group	Medium score group	High score group	
GAD-7	9.65 ± 3.61	12.34 ± 4.18	13.42 ± 5.24	26.159^**^	12.04 ± 4.25	13.81 ± 4.65	13.11 ± 4.26	6.044^**^
PHQ-9	11.73 ± 4.14	14.85 ± 5.23	16.31 ± 6.73	24.331^**^	14.44 ± 5.52	16.47 ± 5.57	15.61 ± 5.47	4.937^**^

PHQ-9 is the abbreviation of patient health questionnaire-9, GAD-7 is the abbreviation of generalized anxiety disorder scale-7.

^**^p < 0.05.

**Table 9:** Comparison of external factors between the low sexual quality of life group and the high sexual quality of life group.

Variables	Male version					Female version				
	Low sexual quality of life group (≥68)	High sexual quality of life group (<68)	*t*	OR	95% CI	Low sexual quality of life group (≥58)	High sexual quality of life group (<58)	*t*	OR	95% CI
GAD-7	13.44 ± 5.23	11.67 ± 4.18	4.37^**^	1.09	[1.05, 1.13]	13.80 ± 4.50	12.79 ± 4.31	2.64^**^	1.05	[1.01, 1.09]
PHQ-9	16.24 ± 6.70	14.11 ± 5.14	4.13^**^	1.06	[1.04, 1.09]	16.62 ± 5.48	15.22 ± 5.47	2.98^**^	1.05	[1.02, 1.08]

OR is odds ratio, CI Confidence interval.

^**^p < 0.05.

## Discussion

This study used a combination of LPA and ROC analysis to determine the optimal cut-off points (58 and 68) for the female and male versions of SQoL-CHD, and verified the effectiveness of the optimal cut-off points in distinguishing between high and low sexual quality of life groups. The research results provide new evidence support for accurate screening, early identification and optimization of clinical intervention strategies for sexual quality of life.

### The scientificity of the optimal cut-off point

This study used LPA to identify latent classes of sexual quality of life among patients with coronary heart disease. The results showed that both female and male patients were divided into three profiles: low, medium, and high groups. The model fit indices and scree plot results supported the optimality of the three-profile model, with Entropy values of 0.962 (female) and 0.969 (male), indicating high classification clarity. The Cohen's *d* values between the profiles were all >0.8, showing significant differentiation (36). On this basis, ROC analysis further determined that the cut-off point for the female version was 58 (AUC = 0.997, 95% CI: 0.995 −0.999, Youden index = 0.943) and the cut-off point for the male version was 68 (AUC = 0.999, 95% CI: 0.998–0.999, Youden index = 0.977). The sensitivity and specificity of both versions exceeded 96%, and the AUC was close to 1. The results showed that the cut-off point had good statistical accuracy and reliability (37).

LPA revealed the heterogeneity of sexual quality of life in patients with CHD, and ROC analysis quantified the predictive power of the cut-off point. The combination of LPA and ROC analysis not only enhances the scientific rigor of setting the cut-off point, but also solves the problem of determining the critical value of the questionnaire score only by subjective experience. This method is relatively reliable in psychometric measurement and risk classification (22, 23, 38, 39). In addition, based on the differences in anxiety and depression levels between the high sexual quality of life group and the low sexual quality of life group divided by the optimal cut-off point, it was found that the anxiety and depression levels of the low sexual quality of life group were significantly higher than those of the high sexual quality of life group. The rationality of the cut-off point was further verified through external validation, which proved that the cut-off point had good discrimination ability. The sufficient sample size of this study ensured the robustness of the results and met the requirements of LPA stability (27). In general, the definition of the cut-off point has high reliability and discriminant validity in psychometrics.

### The practical value of the optimal cut-off point

Sexual quality of life is not only related to the subjective well-being, including aspects such as life satisfaction (40), but also reflects their overall health status, such as heart function, medication reaction, and psychological state (41–43). The results showed that when the score of the female version of the questionnaire was ≥58 or the score of the male version of the questionnaire was ≥68, the patients were divided into the low sexual quality of life group. Compared with the high sexual life quality group, the scores of patients in the low sexual life quality group in the three dimensions of sexual physiology, sexual psychology and sexual cognition were significantly increased, which indicates that the overall sexual life experience of patients with CHD is negatively affected. It is worth noting that 43.3% of male patients and 44.1% of female patients in this study had a disease course of <6 months, suggesting that sexual quality of life problems may emerge in the early stages of CHD (44, 45). Therefore, clear cut-off points provide an important basis for early identification of patients whose sexual quality of life is affected. Medical staff can use this tool to quickly screen high-risk groups in daily assessments, and then implement more targeted health guidance, sexual rehabilitation interventions or psychological support, which will help reduce the risk of adverse adaptation after illness and promote the comprehensive physical and mental recovery of patients. In addition, sex is often seen as a sensitive topic in traditional cultural contexts, and many patients are too ashamed to ask for help (46). In this context, the cut-off points proposed in this study not only improve the objectivity of identifying sexual life problems, but also provide a practical basis for effective communication between doctors and patients, thereby reducing communication barriers and promoting timely detection and intervention of sexual life quality problems in patients with CHD.

### The close association between sexual quality of life and mental health

The study found that low sexual quality of life is closely associated with a higher risk of anxiety and depression. In the three profiles divided by LPA, as the quality of sexual life decreased, the anxiety and depression scores showed an increasing trend, and the mental health risk of the low sexual life quality group was higher than that of the high sexual life quality group, which is consistent with some research results (43, 47–50). Steinke et al. (43) pointed out that the decreased sexual life quality in patients with CHD is often accompanied by psychological distress, and their anxiety scores are significantly higher than those in the normal population. Research by Sobczak et al. (47) demonstrated that patients with CHD with interrupted sexual activity typically exhibit higher anxiety and depression scores, and their mental health was significantly worse than that of patients who maintained their sexual activity. Günzler et al. (50) found that low sexual quality of life was significantly associated with depression levels, reinforcing the perspective of sexual quality of life as an indicator of mental health.

Notably, male with poor sexual activity had a higher risk of anxiety and depression than female, suggesting that intervention design should consider gender differences. The possible reason is that male patients are more likely to have a stronger psychological stress response due to sexual dysfunction and social role pressure, while female patients are more likely to have negative emotions due to lack of emotional connection or relationship distress (51). So, it is recommended to adopt intervention strategies tailored to different genders in clinical interventions and integrate multidisciplinary resources such as cardiac rehabilitation, psychotherapy, and sexual health education to enhance the specificity and effectiveness of interventions.

## Limitations

This study employed a combined approach of LPA and ROC analysis to conduct a data-driven stratification of sexual quality of life in patients with coronary heart disease, providing a replicable analytical method and a reference framework for future research. However, this study still has some limitations. Firstly, this study adopted a cross-sectional design, which limits the ability to determine causal relationships between sexual quality of life and anxiety or depression in patients with coronary heart disease. Low sexual quality of life may contribute to worsening psychological symptoms, and vice versa. In the future, longitudinal studies can be conducted to track the dynamic changes of these variables to clarify the causal direction. Second, the samples of this study were from a hospital in Henan Province, China. The geographical and cultural background may limit the generalizability of the results. Subsequent studies need to validate the applicability of the cut-off points in multicultural populations. In addition, although the questionnaire covers multiple dimensions, it does not directly measure social factors such as partner relationships, which may also significantly influence sexual quality of life. This study primarily focused on core self-reported measures directly aligned with the study objective (sexual quality of life, anxiety, and depression), and therefore did not include additional social or relational variables. Future research could incorporate additional variables, such as partner satisfaction and social support, to improve the model and enhance the interpretability of the cut-off points.

## Conclusion

This study determined the cut-off points of the female and male versions of SQoL-CHD through LPA and ROC analysis, which were 58 and 68, respectively, and validated the effectiveness of the cut-off points in distinguishing high and low sexual quality of life. The determination of these cut-off points has a certain scientific basis and reliability, providing a basis for the screening and intervention of sexual quality of life in patients with coronary heart disease. In the future, this tool can be further optimized and its stability and predictive validity can be verified based on larger samples, multicenter or longitudinal designs, to promote the widespread application of SQoL-CHD in different cultural and population backgrounds, and ultimately help improve the overall quality of life and mental health of patients with coronary heart disease.

## Data Availability

The raw data supporting the conclusions of this article will be made available by the authors, without undue reservation.
